# Artificially designed recombinant protein composed of multiple epitopes of foot-and-mouth disease virus as a vaccine candidate

**DOI:** 10.1186/s12934-017-0648-2

**Published:** 2017-02-22

**Authors:** Ho-Bin Lee, Da-Chuan Piao, Jun-Yeong Lee, Jae-Yun Choi, Jin-Duck Bok, Chong-Su Cho, Sang-Kee Kang, Yun-Jaie Choi

**Affiliations:** 1 Department of Agricultural Biotechnology, Seoul National University, Seoul, 115-921 Republic of Korea; 2 Institute of Green-Bio Science and Technology, Seoul National University, 1447-1 Pyeongchang-Daero, Daehwa-Myeon, Pyeongchang-Gun, Gangwon-Do 25354 Republic of Korea; 3 Research Institute for Agriculture and Life Science, Seoul National University, Seoul, Republic of Korea

**Keywords:** Artificial recombinant protein, B cell epitope, FMDV, GH loop, Multi- epitope

## Abstract

**Background:**

Concerns regarding the safety of inactivated foot-and-mouth disease (FMD) vaccine have been raised since it is produced from cultured live FMD virus (FMDV). To overcome this issue, recombinant protein has been studied as an alternative vaccine.

**Results and conclusion:**

We designed a chimerical multi-epitope recombinant protein (5BT), which is comprised of tandem repeats of five B cell epitopes (residue of VP1 136–162) derived from different FMDV variants and one T-cell epitope (residue of 3A 21–35). To increase solubility and stability of 5BT, it was conjugated with BmpB, the membrane protein B of *Brachyspira hyodysenteriae* (B5BT). Our results indicated that 5BT was susceptible to degradation by host protease and produced with substantial fraction of inclusion body. The stability and solubility of 5BT was greatly increased by conjugating to BmpB. FMDV specific antibodies were observed in the serum of mice immunized with 5BT and B5BT comparable to inactivated FMD vaccine. Sera from 5BT and B5BT groups also exhibited high epitope-specific antibody titers in peptide specific ELISA, indicating that all five epitopes are exposed to the B cell receptor for the antibody reaction. Thus the multi-epitope recombinant protein designed in this study may be a potential candidate as an alternative vaccine against FMDV epidemic variants.

## Background

Foot-and-mouth disease (FMD) causes loss of productivity of animals, leads to large-scale economic shock in livestock industries and induces disadvantages in national trade of livestock products as it causes an acute contagious disease to cloven-hoofed animals such as pigs, cattle, and sheep [1, 2]. Although many researchers have tried to prevent FMD, FMD virus (FMDV) is difficult to be eradicated because of its rapid mutation and variation. Seven different serotypes of FMDV (O, A, C, Asia-1, SAT-1, SAT-2, and SAT-3) have been identified, and rapid mutation rate of serotypes derived numerous variants of serotypes [3, 4]. Serotype O is known as the main serotype of FMDV breaking out in East Asia, Middle Asia, Africa and Europe [5].

Vaccination is considered as the only option to control and prevent FMD. Inactivated virus vaccine for the prevention of FMD has been commercialized [6]. However, it is expensive because the production of inactivated vaccine requires a high level of biological safety facility to prevent the risk of leakage of live virus, and a long time to adapt the virus to cells. The inactivated vaccine is produced by only using the structure proteins (SPs) and removing the non-structure proteins (NSPs) of FMDV, which can be obtained from killing the virus through chemical treatments. If the NSPs are not completely removed in the process, it would cause a serious biosafety concern, which would hinder efforts to employ serology to distinguish between infected and vaccinated animals (DIVA) [7, 8]. This fact leads to classifying all countries as FMD free with or without the use of vaccination by OIE, world organization for animal health. For this reason, most of countries have introduced a policy of the stamping out of FMD infected animal to remain FMD free rather than employing vaccination [2].

Subunit vaccines that consist of recombinant proteins produced in bacteria have been suggested as an alternative to solve these problems [6]. These vaccines are free from DIVA biosafety concern and easy for mass production. However, subunit vaccines with fixed amino acid sequence may have limited efficacy for certain FMD strains because of high mutation rates. For this reason, antibodies produced by existing subunit vaccines have low specificity for neutralizing the mutated FMDV [9]. To overcome this weakness, many researchers have tried to produce newly designed recombinant subunit vaccines which are more effective to FMD [10].

We designed a multi-epitope FMD vaccine candidate composed of five B-cell epitopes and one T-cell epitope to address this variation problem. The technology for construction of multi-epitopic proteins and their usage as vaccines has been already disclosed in a series of patent applications [11, 12]. In addition, several studies already showed that B-cell epitopes are important to produce neutralizing antibodies and T-cell epitopes are also necessary to enhance the immune response by activating T cells to develop a more efficient vaccine against FMDV [13–15]. GH loop (commonly known as amino acid residues 130–160) in VP1 of FMDV is a representative B-cell epitope containing RGD motif, which is an essential sequence to bind integrin of host animal cells for infection [13, 16, 17]. RGD motif region is conserved in most FMDV variants although the rest regions of GH loop are highly variable [4]. We selected five representative GH loop as B-cell epitope (amino acid residue 136–162) among the epidemic strains existing throughout the world for wide protection against various FMDV variants considering its hyper-variability. We also introduced T-cell epitope (amino acid residue 21–35 of 3A protein) in one of FMDV NSPs in *C*-terminus of the protein to enhance the immune response of the subunit vaccine. The subunit vaccine composed of only B-cell epitopes is inadequate to induce an immune response because B cells activated by B-cell epitope need to be stimulated by cytokine secreted from T cells to differentiate into plasma cells for producing antibodies. Blanco et al. proved that the T-cell epitope of 3A protein conjugated in *C*-terminus of the protein enhanced the immune response [18].

There are several huddles to producing artificial recombinant proteins in a soluble form using *Escherichia coli* system. Recombinant proteins expressed in *E.coli* often form inclusion bodies and, in some cases, are not accumulated [19]. The use of fusion partner is a common method to overcome this problem. Recombinant proteins have improved the solubility and stability with the conjugating fusion protein [19, 20]. We introduced membrane protein B of *Brachyspira hyodysenteriae* (BmpB) which caused swine muco-hemorrhagic dysentery, as a fusion partner of multi-epitope subunit vaccine in *N*-terminus of the recombinant protein [21].

In this study, we designed a multi-epitope FMDV subunit vaccine composed of five different B-cell epitopes from five FMDV type O variants conjugated to one T-cell epitope at *C*-terminus. To enhance the solubility and stability of this artificial peptide. BmpB was conjugated with *N*-terminus of the protein. Our results in this study will provide the strategic insight for cost-effective, easy handling, and wide spectrum FMDV subunit vaccine design.

## Methods

### Design of multi-epitope FMD vaccines

**Fig. 1:**
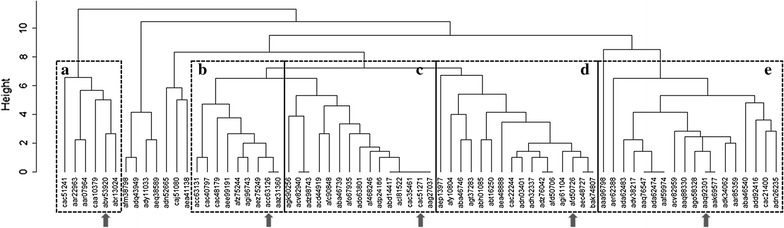
Phylogenetic tree via the correlation analysis of seventy-one GH loop sequence (reside 136–162) from FMDV type O VP1 protein. Height, *y axis* means the number of different amino acids among GH loops. *Open square boxes* mean variant *cluster* and *arrows* indicate five sequences incorporated in 5BT design

Seventy-one peptide sequences of GH loop (residues 136–162) of VP1 were collected from NCBI database, and hierarchical clustering for analyzing the amino acid sequence through R software (Fig. 1) was performed. The final selection was conducted to include one representative GH loop sequence from each major cluster in the phylogenetic tree. A T-cell epitope (amino acid residues 21–35) was selected from 3A of type O FMDV (O-UKG 11/01) [18]. A 504 base pair (bp) synthetic gene, 5BT, which consists of five B-cell epitopes and one T-cell epitope in tandem array, was synthesized in pIDTSMART-AMP (IDT, CA, USA). This gene contains two Xho I restriction sites. To minimize interference between adjacent epitopes, each epitope was separated by two glycines, and T-cell epitope was separated from five B-cell epitopes by two glycines and one glutamate. 5BT gene was cut out by Xho I and ligated with pET21a-BmpB precut with Xho I [21] resulting in pET21a-BmpB-5BT (B5BT). 5BT gene was amplified by PCR from the pIDTSMART-AMP using an upstream primer engineered to introduce an Nde I site (5′- AATTTTACCATATGGGTGGGAGTTATGGCAA ATCCCC-3′) and a downstream one with a Xho I site (5′-GATCCGCTCGAGTTTGATGGACGG -3′). PCR product was cloned into Nde I and Xho I of pET21a precut with the same enzymes, resulting in pET21a-5BT. The recombinant plasmids were confirmed by DNA sequencing at the National Instrumental Center for Environmental Management (NICEM, Seoul, Korea).

### Protein expression and purification

The vectors were transformed into *E. coli* BL21 (DE3) (Novagen, CA, USA) using heat-shock transformation at 42 °C. And, 7 ml of overnight culture was inoculated in 1 L of Luria–Bertani (LB) broth containing 100 ng/ml of ampicillin in 2.8L Fernbach flask. Cultures were agitated at 230 rpm until A_600_ reached 0.6 and expression was induced with 1 mM isopropyl-β-d-thiogalactopyranoside (IPTG) for 4 h at 37 °C. Cells were harvested by centrifugation at 6500 rpm for 10 min at 4 °C. Cell pellets were resuspended in 100 ml of binding buffer (500 mM NaCl, 5 mM imidazole, 20 mM Tris–HCl, pH 7.9) and sonicated on ice (48 × 10 s). Lysates were centrifuged at 17,000 rpm at 4 °C for 20 min and supernatants (soluble fraction) were filtered through a 0.45 µm filter (Corning, NY, USA). 100 ml of binding buffer was added to soluble fraction to purify two target proteins, 5BT and B5BT. The 5 ml bed volume of Ni-nitrilotriacetic acid (NTA) agarose resin (Novagen, CA, USA) was packed into a column and equilibrated with binding buffer. The sample was loaded into a column and the column was washed with 20 resin volume of binding buffer followed by 10 resin volume of wash buffer (40 mM imidazole, 0.5 M NaCl, 20 mM Tris–Cl, pH 7.9). Target protein was eluted with 20 ml of elution buffer (1 M imidazole, 0.5 M NaCl, 20 mM Tris–Cl, pH 7.9). The eluted protein was dialyzed using a membrane tube (molecular cut-off: 6–8000 kDa, Spectrum, CA, USA) against the distilled water at 4 °C overnight. Desalted solution was lyophilized and stored at −20 °C until used. Lipopolysaccharide (LPS) was removed using ToxinEraser™ Endotoxin removal kit (Genscript, NJ, USA) and detected by using ToxinSensor™ Chromogenic LAL endotoxin assay kit (Genscript, NJ, USA). OD_280_ was detected by a spectrophotometer (Implen, Munchen, Germany) and protein concentration was calculated using extinction coefficient [22]. To analyze the inclusion body formation, sonicated cell debris was dissolved in 100 ml of solubilization buffer (10 mM tris-base, pH 12.5) and centrifuged at 17,000 rpm at 4 °C for 20 min. Supernatant containing dissolved inclusion body (inclusion body fraction) was transferred to other tubes.

### Analysis of solubility and stability of recombinant proteins

The 20 µl of soluble and inclusion body fractions were analyzed by 15% sodium dodecyl sulfate–polyacrylamide gel electrophoresis (SDS–PAGE). The gels were stained with Coomassie Brilliant Blue by 3 times of heating in a microwave oven for 70 s, cooled down on a rocker for 5 min and destained with 25% methanol and 7.5% acetic acid solution overnight. Bands were analyzed by image J software (NIH) to compare target protein quantity [23]. The target protein was confirmed by western blot assay using His-tag antibody (Abcam. MA. USA). The protein was separated in a 15% SDS–PAGE and then transferred to a nitrocellulose membrane (Whatman, Germany). The membrane was blocked by 5% skim milk in tris buffered saline (TBS) contacting 0.05% Tween 20 (TBST) for 1 h on a rocker and then washed three times with TBST. The membrane was incubated with a 1:1000 diluted his-tag antibody overnight at 4 °C, washed three times with TBST, and incubated with a 1:2000 dilution of rabbit anti-mouse IgG antibody conjugated with horseradish peroxidase (HRP) (Abcam, MA, USA) for 1 h. After washing three times with PBST, the signal was developed tetramethylbenzidine (TMB). To test stability of the proteins, the cell pellets from 50 ml culture were resuspended in 10 ml of PBS and distributed in 1 ml aliquot into the micro tube. The tubes were centrifuged at 13,000 rpm for 1 min at ambient temperature. The supernatant were removed and cell pellets were stored at −70 °C until use. Every day one frozen tube was resuspended in 1 ml of PBS, sonicated and supernatants after centrifugation were stored at 4 °C. This was repeated for 7 days to investigate the protein degradation by endogenous proteases of *E. coli*. After 7 days proteins were analyzed by 15% SDS–PAGE, bands of target proteins in gel images were analyzed by Image J software.

### Mouse immunization

Six-week old BALB/C mice were used for the immunization following the policy and regulations for the care and use of laboratory animal (Laboratory Animal Center, Seoul National University, Korea). All of the protocols were reviewed and approved by the Animal Care and Use Committee at Seoul National University (SNU-141201-1). The mouse was immunized intramuscularly at days 0, 14 and 28 with 20 µg (0.5 µg/µl) of each peptide emulsified in Complete Freund`s Adjuvant (CFA, priming) or Incomplete Freund`s Adjuvant (IFA, boosting) and sacrificed on day 42. Five mice in the negative control group were immunized with PBS and positive control group of 5 mice were immunized with 40 µl of inactivated FMDV vaccine (iFMDV, Daesung, Gyeonggi-do, Korea). Blood samples were collected before priming (day 0) and on days 13, 27, and 42 (Sacrifice) from intra-petrosal veins with a disposable syringe and delivered into sterilized tube. Serum was separated by centrifugation at 12,000 rpm for 3 min using serum separate tube (BD microtainer, NJ, USA).

### ELISA assay

Antibody production was examined by ELISA in serum samples collected at days 0, 13, 27, and 42. Briefly, 96 well immuno-plate was coated with purified 5BT in carbonate-bicarbonate buffer (CBB) for 1 h at 37 °C (0.1 µg/well) or to evaluate peptide specific antibody production about five B cell epitopes in the 5BT were separately synthesized (Peptron, Daejeon, Korea) and dissolved in DMSO. Plates were coated with 50 ρmole/well of each peptide in the CBB. Then, wells were washed with PBS and blocked with 0.5% skim milk in PBS for 1 h at room temperature (RT). Series of fivefold dilution of each serum sample were prepared, starting at 1/50 and volume adjusted to 100 µl with PBST (0.5% tween 20 in PBS) containing 0.5% skim milk. Plates were incubated for 2 h at RT and HRP conjugated goat anti-mouse antibody diluted 1:5000 in PBST containing 0.5% skim milk was added. The color was developed with 100 µl/well of the TMB (Sigma, MO, USA) and stopped by an equal volume of 0.16 M H_2_SO_4_. Plates were read at 450 nm in a Microspectrophotometer (Tecan, Austria). Titer of specific antibody was calculated by Softmax Pro version 5.4.1. Antibody titers were reported as log10 of the reciprocal of the highest dilution. Serum of days 0, 13, and 27 were analyzed with above methods according to time by detecting 5BT specific IgG titers.

In addition, anti-FMDV O type antibodies were detected by competition ELISA using VDPro FMDV type O ELISA kit (Median diagnostics, Gangwon-do, Korea), following the manufacturer’s protocol. Briefly, each plate of the kit was pre-coated with FMDV type O P13C protein. Serum sample, negative control, and positive control were diluted by 1:5 in dilution buffer and prepared samples were incubated in wells for 1 h at RT. Then, wells were washed with washing buffer, 100 µl of HRP conjugated anti-FMDV antibody was added and samples were incubated for 1 h at RT. Color was developed with 100 µl/well of the TMB substrate and stopped by 50 µl of stop solution. All reagents were provided in the kit. Plates were read in a Microspectrophotometer at 450 nm. PI (%) means the percent inhibition [24].

### Statistical analysis

Statistical analysis was performed using OriginPro 9.0 software (OriginLab, USA). For the significance of tests, a one-way analysis of variance (ANOVA) followed by Tukey’s post hoc test was used, and expressed as follows; *P < 0.05, **P < 0.01, ***P < 0.001.

## Results

### Design of the recombinant peptides and vector construction

**Fig. 2:**
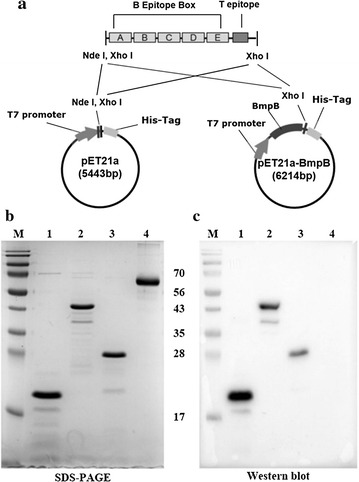
**a** Schematic diagram for construction of recombinant proteins expression vector system. **b** SDS–PAGE gel stained with *Coomassie Brilliant Blue*. Each *lane* was loaded with 2 µg of purified recombinant proteins. Lanes: M, proteins marker; 1, 5BT (18.1 kDa); 2, B5BT (45.8 kDa); 3, BmpB (28.7 kDa); 4, BSA (66.5 kDa). **c** Western blot analysis recombinant proteins were detected with His-tag using anti-His-tag antibody. BmpB is a positive control and commercial BSA is a negative control

We gathered sequence information of VP1, one of the structural proteins of FMDV type O, at NCBI GenBank and analyzed the amino acid sequence similarity of various GH loop peptides (amino acid residues 136–162 of VP1) through hierarchical clustering (Fig. 1.). The peptide sequences were classified into five clusters and one representative peptide of each cluster was selected to cover all five clusters of FMDV variants (Table 1). We constructed the recombinant plasmid which ligated the multi-epitope 5BT gene consisted of the selected five GH loops (B-cell epitopes) and one T-cell epitope into pET21a and pET21a-BmpB for expression of 5BT and B5BT, respectively (Fig. 2a). The expressed proteins were purified by his-tag purification (Fig. 2b). The peptides except BSA were detected by western blotting assay using anti-His-tag antibody (Fig. 2c). The concentration of the acquired proteins were measured with the previously described method, protein extraction and purification [25]. It was determined that purified 5BT was produced by 42 mg per litter culture and purified B5BT was produced by 11.6 mg per litter culture.

**Table 1:** Information of B cell epitopes and T cell epitope used in this study

No	Year	Accession number	Country	Peptide sequence	Note
A	2000	abv53920	China	YGKSPVTNLRGDLQVLTQKAARTLPTS	VP1
B	1963	acc63126	Belgium	YSRNAVPNLRGDLQVLAQKVARTLPTS	VP1
C	2000	cac51271	Korea	YGESPVTNVRGDLQVLAQKAARTLPTS	VP1
D	2010	afd50726	Korea	YAGGSLPNVRGDLQVLAQKAARPLPTS	VP1
E	2010	aaq92301	Kenya	YGRAPVTNVRGDLQVLAQKAARTLPTS	VP1
F	2001	abu63090	England	AAIEFFEGMVHDSIK	3A

Outbreak nation, year, NCBI accession number of FMDV and amino acid sequences included in 5BT design

### Characterization of proteins

**Fig. 3:**
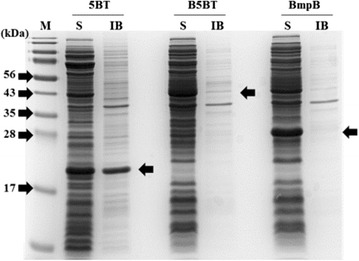
SDS–PAGE analysis for the expression pattern of recombinant proteins. Lanes: M, protein marker; S, soluble crude protein; IB, crude inclusion bodies; 5BT, 18.1 kDa; B5BT, 45.8 kDa; BmpB, 28.7 kDa. The stained gel was analyzed by image J software and inclusion body fractions were: 5BT, 36%; B5BT, <2%; BmpB, <1%

**Fig. 4:**
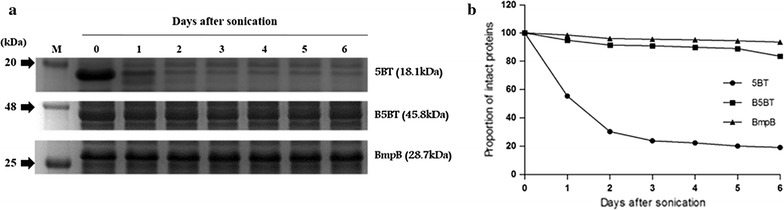
BmpB effect on the stability of recombinant proteins. **a** SDS–PAGE gel stained with *Coomassie Brilliant Blue*. Crude protein extracts were incubated at 4 °C for 6 days and daily sample was analyzed by SDS–PAGE. Lanes: M, protein marker. **b** Graph showing intact proteins in (**a**) analyzed by image J software

To examine the effect of BmpB as a fusion partner, the soluble and insoluble fractions of 5BT, B5BT and BmpB were prepared and analyzed by SDS–PAGE (Fig. 3a). The previous report showed that BmpB was expressed fully as a soluble protein [21]. The ratio of soluble and inclusion body fraction were analyzed by image J software (Fig. 3b). 5BT protein was produced mostly in the soluble form. Although 5BT were mostly produced in soluble form, approximately 36% of 5BT were produced as the inclusion bodies. We introduced fusion partner, BmpB, into 5BT to improve the solubility of protein. B5BT conjugated BmpB in *N*-terminus of 5BT was expressed as 98% of soluble protein. Though 5BT was expressed into soluble protein by introducing B-cell epitope containing secondary structure sequence, most of other 5BT region was unstructured in native VP1 and thus, a potentially unstable structure was predicted. As predicted, the 5BT proteins were degraded when incubated the crude protein extracts at 4 °C (Fig. 4a). 5BT rapidly degraded with 45% loss in 24 h whereas BmpB and B5BT were stable for at least 6 days (Fig. 4b). Thus BmpB as a fusion partner increased the solubility of 5BT and protected the artificial proteins against endogenous host proteases.

### Evaluation of multi-epitope 5BT and B5BT as antigens

**Fig. 5:**
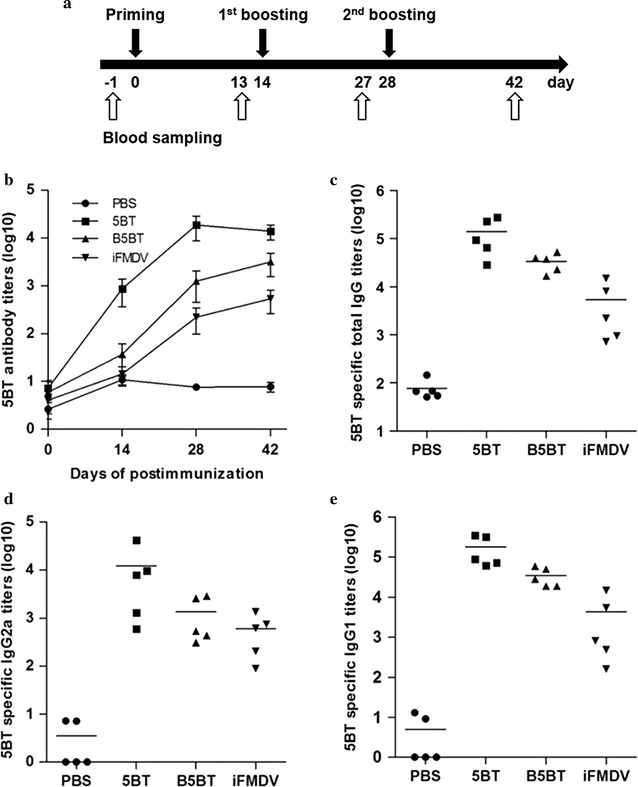
Validation of recombinant proteins as FMDV subunit vaccine in vivo. **a** Schematic view of immunization and blood sapling schedule (n = 5/group). **b** Antibody titer analysis by ELISA. Specific antibody titers against 5BT was measured by ELISA in serum samples collected at days 0, 13, 27 and 42 post-immunization. Antibody titers were expressed as the reciprocal log10 of the last dilution calculated by interpolation to give an absorbance of 1 above background. Each point corresponds to the geometric mean of each groups. *Error bars* represent standard error of the mean. Validation of immune response route of recombinant proteins. **c** 5BT specific total IgG titers detected by ELISA at day 42 post-immunization, **d** IgG1, and **e** IgG2a. Endpoint titers were expressed as the reciprocal log10 of serum dilutions. Each *symbol* represents the value of individual mouse. *Horizontal lines* indicate the mean of each group of animals

The immunogenic effect of 5BT and B5BT as subunit vaccines was tested in mice via the intramuscular injection. PBS and inactivated FMD vaccine (iFMDV) were used as a negative and a positive control. 5BT specific antibodies were determined by ELISA in the serum collected at days 0, 13, 27 and 42 after priming immunization (Fig. 5a, b). 5BT specific antibodies increased in 5BT, B5BT, and iFMDV injection group. 5BT specific antibody titer was the highest in 5BT group compared to B5BT or iFMDV group which has the lowest one.

Figure 5 showed that antibodies produced in the group injected iFMDV, vaccine produced with MANISA O1 strain, recognized 5BT although it does not contain GH loop peptide of MANISA O1. Furthermore, BmpB fusion did not affect the production of 5BT specific antibodies in B5BT group. To evaluate the route of immune response, total IgG and IgG subtype (IgG1 and IgG2a) titers were determined using day 42 (Fig. 5c–e). All treatment groups induced the balanced immune response of IgG1 and IgG2a, which indicates the balanced activation of Th1 and Th2 route.

### Evaluation of multi-epitope 5BT and B5BT as subunit vaccine

**Fig. 6:**
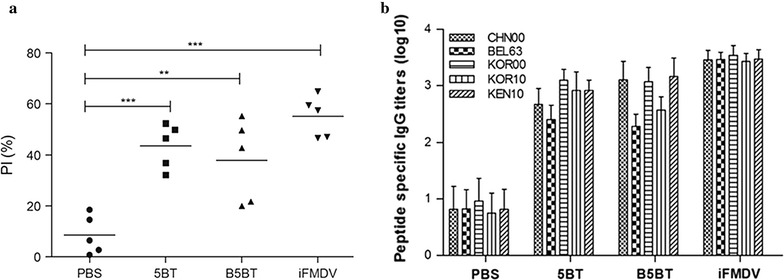
**a** Detection of FMDV specific antibody response in serum analyzed by competition ELISA assay at day 42 post-immunization. The PI (%) means the percent inhibition. [PI = 100–100 × (OD_450_ of sample serum/OD_450_ of negative control)]. Negative and positive controls were satisfied with *standard recommending* of manufacturer manual. Each *symbol* represents the value for individual animals.* Horizontal lines* describe the mean value for each group of animals. **: P < 0.01; ***: P < 0.001, one way ANOVA. **b** Antibody response to each peptide in 5BT in serum analyzed by ELISA assay at day 42 post-immunization. Antibody titers were expressed as the reciprocal log10 of the last dilution calculated by interpolation to give an absorbance. *Error bars* represent standard deviation

Anti-FMDV type O antibodies were evaluated using a liquid phase block (LPB) ELISA (Fig. 6a). Structure proteins originated from MANISA O1 strain (iFMDV) were coated on the wells of the immuno-plate for competition ELISA. Antibodies in the serum of three groups were significantly higher compared to the PBS group. Although FMD vaccine contained more epitopes other than GH loop compared to 5BT proteins, there was no significant difference between the iFMDV and 5BT or B5BT group in this competition assay. Sera of 5BT and B5BT group successfully compete with commercial antibodies that are bound to VP1 protein of FMDV in competition ELISA.

The accessibility of B-cell receptors was estimated using an ELISA to each GH loop peptide of 5BT for the production of epitope specific antibody (Fig. 6b). If epitopes are exposed on the surface of a protein, it may lead to higher B-cell specific antibodies than those buried inside the protein. All epitopes of 5BT lead to produce their specific antibodies. Sera from 5BT and B5BT groups showed the similar peptide specific antibody titers with anti-BEL 63 antibodies being the lowest among the groups. Sera of iFMDV group reacted very similarly to all GH loop peptides of 5BT.

## Discussion

The shortcomings such as high production cost, safety issue, and low protection rate due to high mutation rate of FMDV of chemically inactivated vaccine have been discussed to prevent FMD for a long time. Various strategies were brought up to overcome these problems. Subunit vaccine is the safest and the cheapest among them [6]. Especially, production of recombinant proteins in *E. coli* as bioreactor was most popular and the protocols were standardized [26]. The popularity of *E. coli* system comes from relatively inexpensive development procedures, simple cultivation procedures, and easy extraction of recombinant proteins [27].

Many researchers developed subunit vaccines to prevent FMD and showed that multi-epitope vaccine was the best strategy for the livestock industry. [18, 28–30]. This concept has been already developed and publicly disclosed in a series of patent applications [11, 12]. Functions and structure of the proteins consisted of FMDV have been thoroughly studied. VP1, a structure protein, has a GH loop region which binds to integrin of host cells and causes infection of animals. The surface region of VP1 containing GH loop has been related to neutralizing antibodies [31]. VP1 containing the linear and conformational epitope region is a good subunit vaccine candidate comparable to the effect of inactivated vaccine [32, 33]. However, it was reported that whole VP1 was produced in inclusion body form in *E. coli* [34, 35]. Inclusion body was produced by aggregation of miss folded proteins. Aggregated proteins have low bioactivity because it has insoluble character. In this reasons, many researchers have tried to make soluble protein from inclusion bodies using high concentration of urea and acetone precipitation [36–39]. Therefore, solubilization is very important step for the production of subunit vaccine, and the addition of re-solubilizing step increases the production cost.

It cannot be predicted whether the artificial protein 5BT is expressed in soluble or inclusion body form, or not accumulated in the recombinant host cells because this peptide is an artificially designed protein that does not exist in nature. We also designed and tried to express the recombinant protein containing five B cell epitopes covering amino acid residues 132–151. However, this construction was not expressed in *E. coli* (data not shown). There is a classical hypothesis for the mechanism of folding in which secondary structure, such as helices, turns, and sheets, are formed first and then dock to form the tertiary structure [40]. We thought that peptide (amino acid residues 136–162) containing the secondary structure region might help the structural formation of artificially designed protein. It was reported that in VP1 3D structure, GH loop region (amino acid residues 136–162) is mostly un-structured on the surface of VP1 [41], except 8 amino acids (amino acid residues 148–155) following RGD motif which forms alpha-helical structure [13]. Thus, the expression vector was constructed to cover GH loop region of the secondary structure (amino acid residue 136–162 of VP1), and 5BT protein was produced mostly in soluble form.

Though 5BT are mostly produced in soluble form, about 36% of 5BT was produced as the inclusion body. Moreover, 5BT was unstable in crude protein extract and degraded in the production process. In most organism, miss-folded and unstable proteins are degraded by endogenous protease [42, 43]. This makes it complicated to produce and store the recombinant subunit vaccine candidate, e.g. cell harvest and purification steps. To overcome this problem, BmpB was introduced as a fusion protein for the solubility and stability of 5BT. Conjugation of the fusion protein is one of methods frequently used to express soluble protein by improving the stable-structure. The BmpB is known to express in soluble form in large amount in *E. coli*, and the result indicated that BmpB could resist to endogenous protease. In addition, the B5BT will be able to use as a conjugate vaccine for the prevention of FMDV and *Brachyspira hyodysenteriae* that causes swine dysentery. The stability and solubility of 5BT protein were greatly improved by BmpB conjugation although the expression level was lower compared to 5BT alone. However, this can be overcome by controlling culture conditions, culture temperature, induction time, inducer concentration, and harvest time [26]. Furthermore, it may be possible that re-cloning with a codon optimized fusion partner BmpB will improve the expression level, which will perform the experiment in a near future.

From the mouse immunization experiment four results were noteworthy for effects of the artificial proteins. Firstly, 5BT specific antibodies were detected in the sera of 5BT and B5BT groups. B cell recognizes an epitope of antigens with B cell receptors and is activated by cytokines from T cells. Activated B cell differentiates into plasma cells which secrete antibodies. The antibodies bind to the epitope recognized by B-cell receptors [44]. B-cell epitopes should be exposed on the surface of antigen for recognition by B-cell receptors [45]. It is indicated that 5BT region of B5BT is exposed and BmpB does not hinder the epitope access by B cell receptors. Secondly, although 5BT does not have the GH loop sequence of Manisa O1 strain (Table 1), which is the source of iFMDV vaccine, the sera of iFMDV group showed specific binding to 5BT and B5BT probably through the antibodies recognizing the conserved region containing RGD motif. It also suggest that 5BT and B5BT vaccine can protect other strain not included in 5BT through the reaction to this conserved region. Multi-epitope subunit vaccine has an effect of high density epitope to improve chances of recognition from B cell receptor [46]. It is important to prevent a wide spectrum of FMD variants. Thirdly, it was confirmed that immunization with 5BT and B5BT elicited the production of FMDV neutralizing antibodies in mice. Sera of 5BT and B5BT groups successfully completed with commercial antibodies that bound to VP1 protein of FMDV in competition ELISA kit [24]. It was confirmed that immunization with 5BT and B5BT elicited the production of meaningful antibodies such as neutralizing antibodies against FMDV, in animals. Lastly, antibodies from all three antigenic groups showed more or less similar specific binding affinity to each synthetic peptide composed of 5BT. Although exposure of epitope is influenced by protein folding pattern [45], B5BT and 5BT groups showed similar results. It means that BmpB increases the solubility and stability of 5BT without the conformational binding inhibition. In both 5BT and B5BT groups, BEL63 specific antibody had the lowest titers compared to other peptides. 5BT protein is expected to have the secondary structure of the linear and alpha helix repeating five times and a terminal T cell peptide. Kloss et al. suggested insight of protein structures consisting of repeating peptides. Adjacent repeats packed together in a more-or-less linear array, facilitating a simple linear representation of energetics, similar to that of DNA double helix formation. 5BT proteins or 5BT region in B5BT might form single super secondary structure loop [47]. It is suggested that BEL63 epitope in the second position from *N*-terminus of 5BT may be least exposed to the B-cell receptors for the antibody reaction.

## Conclusion

In conclusion, an artificial peptide containing five different epitopes from worldwide FMDV epidemic strains ware designed and successfully expressed in soluble and stable form in *E. coli*. Through immunization of purified protein in mice, the peptide`s potential as a FMD subunit vaccine candidate was verified. The study provides insight about the design and selection of multipotent artificial recombinant protein as a vaccine for a highly mutable viral disease such as FMD. In the future, immunization assay should be performed in cloven-hoofed animals for greater efficacy, and other vaccine development protocols should be employed.
